# A Novel Substance P-Based Hydrogel for Increased Wound Healing Efficiency

**DOI:** 10.3390/molecules23092215

**Published:** 2018-08-31

**Authors:** Da Jung Kim, Ji Hae Jang, Song Sun Jang, Jungsun Lee

**Affiliations:** Research and Development Institute, Biosolution, Seoul Technopark, 232 Gongneung-ro, Nowon-gu, Seoul 01811, Korea; xkssls@naver.com (D.J.K.); kimdj1404@kaist.ac.kr (J.H.J.); xkssls@kaist.ac.kr (S.S.J.)

**Keywords:** substance P, stability, wound healing, hydrogel, topical agent

## Abstract

The neuropeptide substance P (SP) is known to stimulate wound healing by regulating the production of relevant cytokines as well as cell proliferation and migration. However, the therapeutic application of SP is limited by its low stability under biological conditions and oxidation during purification, formulation, and storage. To address this problem, we developed a novel formulation of SP as an SP gel, and investigated its wound healing activity both in vitro and in vivo. SP in SP gel was stable at various temperatures for up to 4 weeks. In vitro, SP gel exhibited more potential as a candidate wound-healing agent than SP alone, as evidenced by the observed increases in the proliferation and migration of human epidermal keratinocytes and human dermal fibroblasts. In vivo experiments showed that SP gel treatment enhanced the healing of full-thickness wounds in mice as compared to SP alone. These results demonstrate the benefits of SP gel as a promising topical agent for wound treatment.

## 1. Introduction

Wound healing is a critical process that follows wounding [1] involving the activation of multiple signaling cascades that stimulate tissue repair [2] as well as inflammation, cell proliferation and migration, and tissue remodeling [3]. These processes are mediated by cytokines and small molecules [4,5]. Among them is an 11-amino acid neuropeptide known as substance P (SP) [6,7,8] that is produced by both neuronal and immune cells during tissue injury and exhibits potent wound healing activity [9] that is exerted via induction of epidermal cell and fibroblast proliferation and angiogenesis [10,11]. Topical application of SP enhanced wound closure in nitric oxide synthase-deficient mice and in diabetic and non-diabetic rats [7,8,12]. SP acts by regulating the production of various cytokines including tumor necrosis factor-alpha (TNF-α) and interleukin-1beta (IL-1β), -2, -6, and -8 as well as growth factors such as vascular endothelial growth factor and transforming growth factor-beta1 (TGF-β1) that are involved in wound healing [7,13,14,15]. However, therapeutic application of SP has been hindered by its low stability; specifically, various proteases (e.g., chymotrypsin and angiotensin-converting enzymes) degrade SP under biological conditions [8,16,17], which can delay wound healing. Additionally, SP is unstable in oxidative reactions that can occur during the purification, formulation, and storage of protein-based pharmaceuticals [18], which reduce shelf life and pharmacological potency over time [19]. Indeed, oxidative degradation was shown to reduce SP activity by up to 3-fold [20]. Therefore, novel formulations of SP with improved stability are needed for more effective wound treatment.

To this end, in the present study we used the antioxidant sodium thiosulfate as well as the surfactant polysorbate 80 to increase the stability of SP. Hydroxyethyl cellulose (HEC), a universal gelling agent, was also included in the new formulation. Our in vitro and in vivo experiments demonstrate that the SP gel had more potent wound healing activity than SP alone, and may therefore have broad clinical applicability.

## 2. Results 

### 2.1. Optimization of the SP Gel Formulation

**Figure 1:**
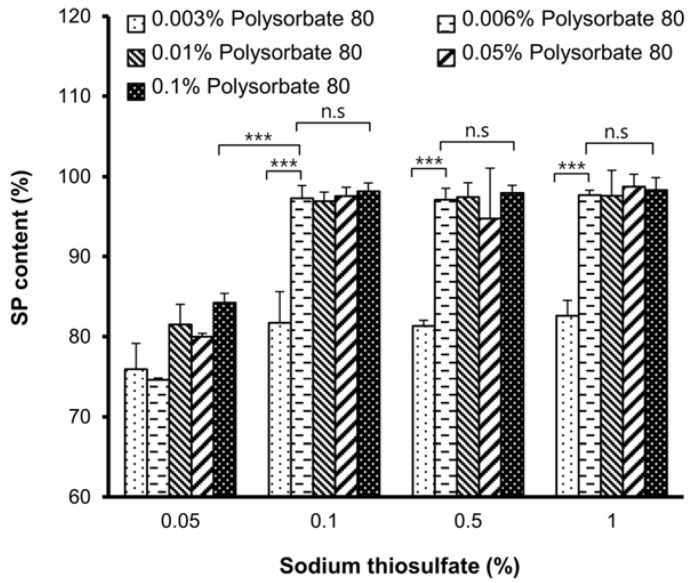
Ratios of sodium thiosulfate and polysorbate 80 in the SP gel formulation. Sodium thiosulfate and polysorbate 80 ratios in SP gel were determined by analyzing the SP content in the 5 μg/mL SP solution containing sodium thiosulfate and polysorbate 80 by ELISA. Values represent mean ± SD of three independent experiments. *** *p* < 0.001.

To improve the stability of SP, an optimized ratio of sodium thiosulfate/polysorbate 80 was determined by analyzing SP content in a solution of 5 μg/mL SP dissolved in PBS with sodium thiosulfate and polysorbate 80 at room temperature for 1 h by ELISA (Figure 1). A significant large decrease (20%) in SP content was observed for the mixture containing 0.05% sodium thiosulfate (*p* < 0.001) or 0.003% polysorbate 80 (*p* < 0.001) relative to the control sample without incubation at room temperature. However, in mixtures containing ≥0.1% sodium thiosulfate and ≥0.006% polysorbate 80, the SP content was nearly 100%, which was same as that of the control. The lowest concentrations of sodium thiosulfate (0.1%) and polysorbate 80 (0.006%) that improved SP stability were selected for the SP gel formulation to minimize cost and avoid the toxic effects of higher concentrations [21]. HEC (1.5%) was added to the mixtures to further increase viscosity and stability, with gelation occurring within 30 min at room temperature (Table 1). An HEC concentration of 1.5% did not cause any obvious damage or elicit an inflammatory response in the mouse model [22].

### 2.2. Analysis of SP Gel Stability at Various Temperatures

**Figure 2:**
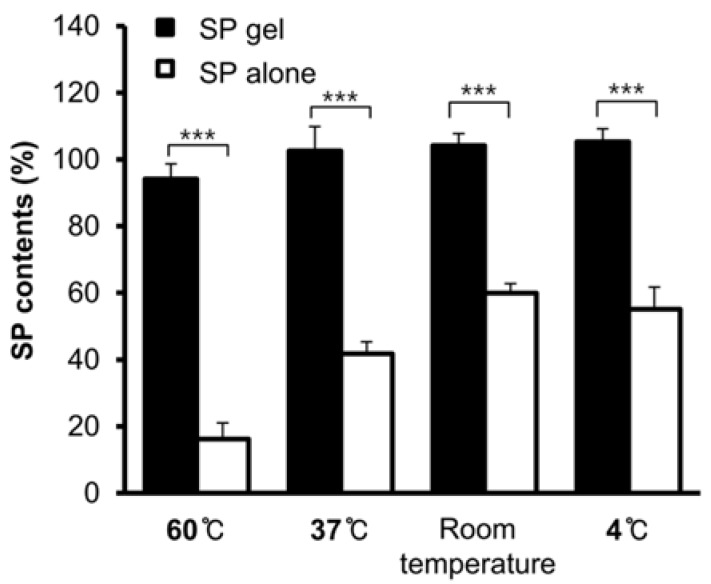
Analysis of SP gel stability at various temperatures. SP alone or SP gel was stored at 60 °C, 37 °C, room temperature, and 4 °C for 4 weeks. A sample at 0 weeks without incubation at different temperatures served as a control. The stability of SP alone and SP gel was analyzed by ELISA. Values represent mean ± SD of three independent experiments. *** *p* < 0.001.

To investigate the stability of SP gel under oxidative conditions, the gel was incubated for 4 weeks at 60 °C, 37 °C, room temperature, or 4 °C. After 4 weeks, the SP content of SP gel at each temperature was ~95% of the value of the 0-week sample (Figure 2). We also performed the same experiment with SP alone and found that in contrast to that in SP gel, SP content was significantly decreased by nearly 20% and 40% by incubation at 60 °C (*p* < 0.001) and 37 °C (*p* < 0.001), respectively. Moreover, after 4 weeks of incubation at room temperature or at 4 °C, the SP content in SP alone was also significantly decreased by nearly 60% (*p* < 0.001), suggesting that SP is inherently unstable at various temperatures over the long term.

### 2.3. Potential of SP Gel as a Candidate Wound-Healing Agent In Vitro 

In vitro proliferation and migration assays were used to evaluate the contribution of SP gel on wound healing. In these experiments, HEC concentration in the SP gel was reduced 100-fold (to 0.015%) to avoid interference with cell growth [22].

**Figure 3:**
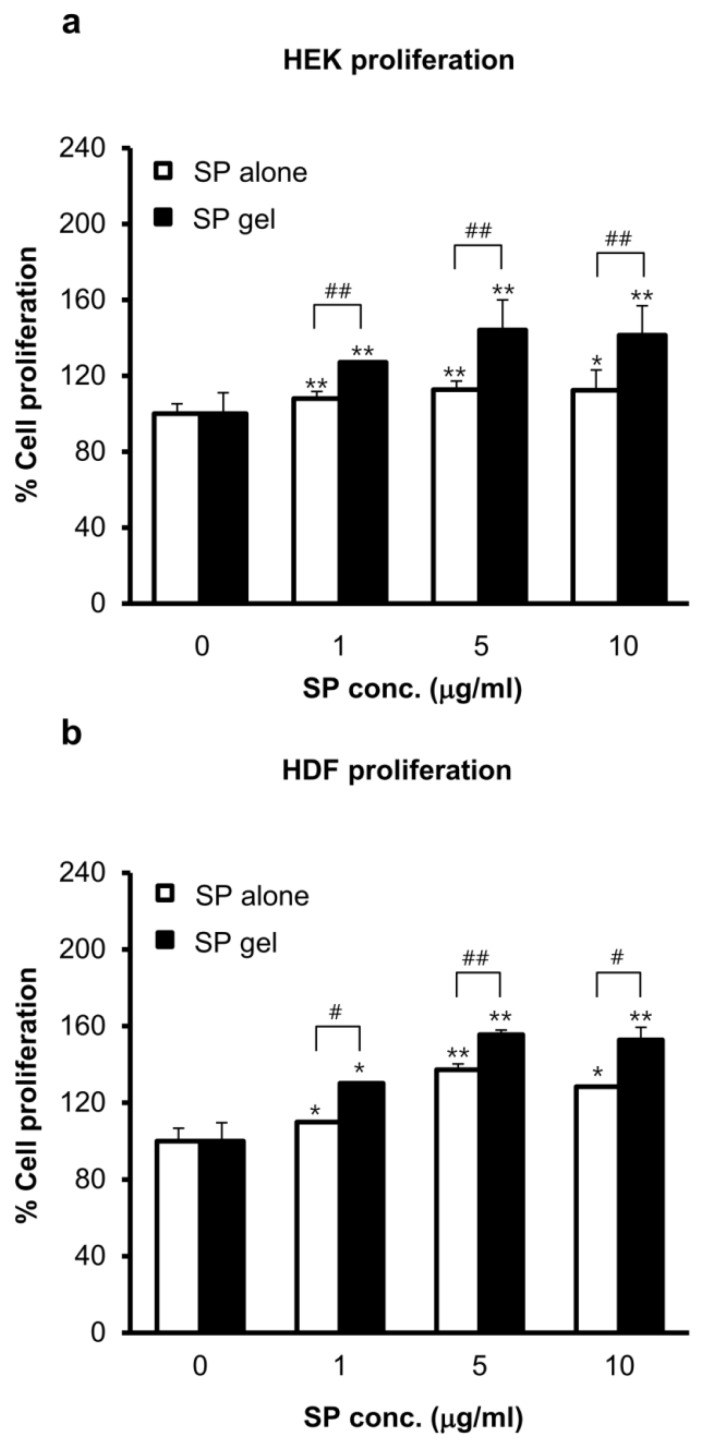
SP gel-induced cell proliferation. (**a**,**b**) HEK and HDF proliferation induced by SP gel. After incubation for 24 h with indicated concentrations of SP alone or SP gel, the viability of HEKs (**a**) and HDFs (**b**) was assessed with the MTT assay. Values represent the mean ± SD of three independent experiments performed in duplicate. * *p* < 0.05, ** *p* < 0.01 vs. PBS-containing medium without SP; ^#^ *p* < 0.05, ^##^ *p* < 0.01 vs. SP alone.

The effect of SP gel on HEK and HDF proliferation was evaluated with the MTT assay. Proliferation of HEKs (Figure 3a) and HDFs (Figure 3b) increased in a dose-dependent manner for SP concentrations ranging from 0 to 10 μg/mL, with optimal proliferation observed at 5 μg/mL SP. In HEKs and HDFs treated with SP gel, a significant difference (*p* < 0.01) in proliferation rate was observed between HEKs and HDFs, as compared to that in medium containing PBS, and the rate was significantly higher than that for the cells treated with only 5 μg/mL SP (*p* < 0.01). Treatment with gel lacking SP had no effect on proliferation rates relative to medium containing PBS only.

**Figure 4:**
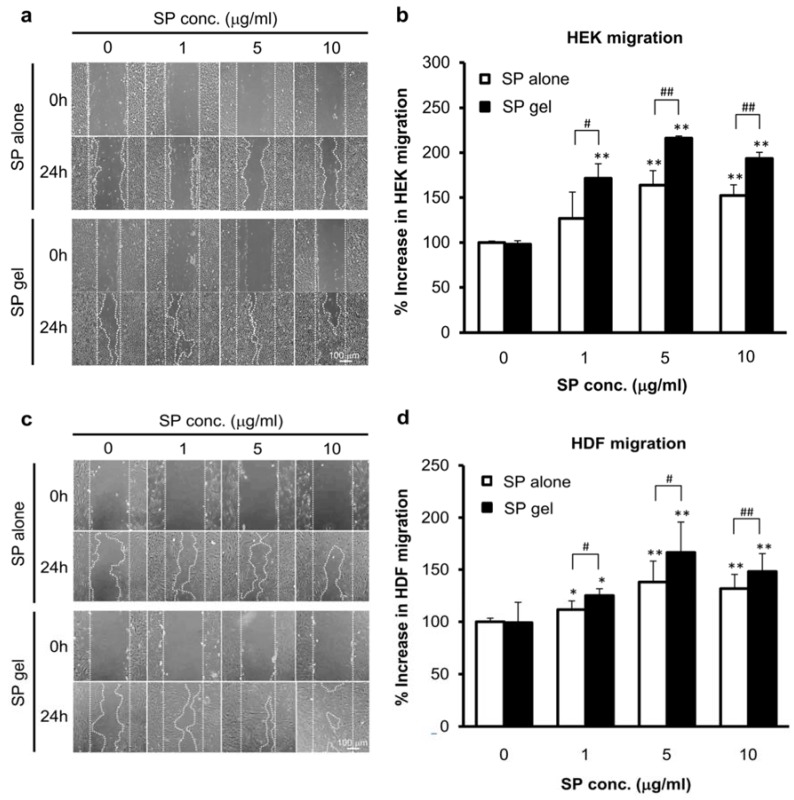
SP gel-induced cell migration. (**a**–**d**) HEK (**a**,**b**) and HDF (**c**,**d**) migration in vitro was assessed by the cell migration assay. Cells were grown to confluence and then treated with SP alone or SP gel; migration was monitored for up to 24 h. Representative images are shown (**a**,**c**). Migration rates were calculated from the micrographs (**b**,**d**). Values represent the mean ± SD of two independent experiments performed in duplicate. * *p* < 0.05, ** *p* < 0.01 vs. PBS-containing medium without SP; ^#^ *p* < 0.05, ^##^ *p* < 0.01 vs. SP alone.

The cell migration assay revealed that migration of HEKs and HDFs into the scratched area was significantly increased in the presence of SP gel as compared to that in PBS-containing medium 24 h making the scratch (*p* < 0.01), with maximum migration observed at 5 μg/mL SP (Figure 4). SP gel was more effective in inducing cell migration than SP alone. Specifically, the migration rate of HEKs and HDFs was 50% (*p* < 0.01) and 30% (*p* < 0.05) higher, respectively, upon treatment with SP gel containing 5 μg/mL SP as compared to that with 5 μg/mL SP in PBS; that is, cell migration was increased by 163% and 137%, respectively, relative to that for PBS-treated control cells upon treatment with SP alone, and by 216% and 167%, respectively, upon treatment with SP gel.

### 2.4. Efficacy of SP Gel for Wound Healing In Vivo

**Figure 5:**
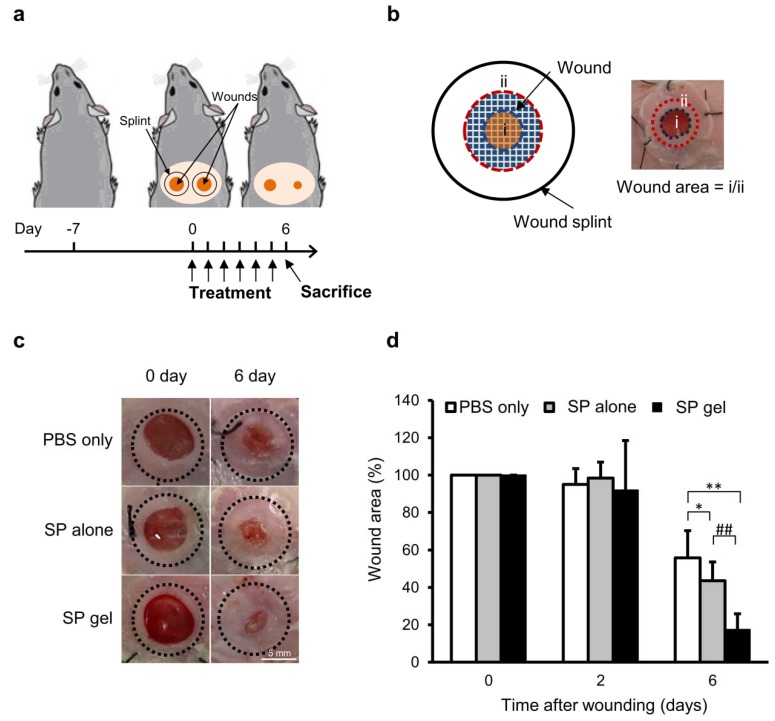
In vivo assessment of the efficacy of SP gel for healing mouse excisional wounds. (**a**) Schematic of the time course of the mouse excisional wound splinting model. (**b**) Relative wound area was calculated as actual wound area (i) divided by splinted hole area (ii). (**c**) Wound closure upon treatment with SP alone, SP gel, or PBS alone was monitored every 24 h. Representative images of wounds on day 0 and 6 are shown. (**d**) Changes in wound area at indicated time points relative to the original area. Values represent mean ± SD (*n* = 13 for PBS alone, *n* = 8 for SP alone, and *n* = 5 for SP gel). * *p* < 0.05, ** *p* < 0.01 vs. PBS-only negative control; ^##^ *p* < 0.01 vs. SP alone (Student’s *t* test).

**Figure 6:**
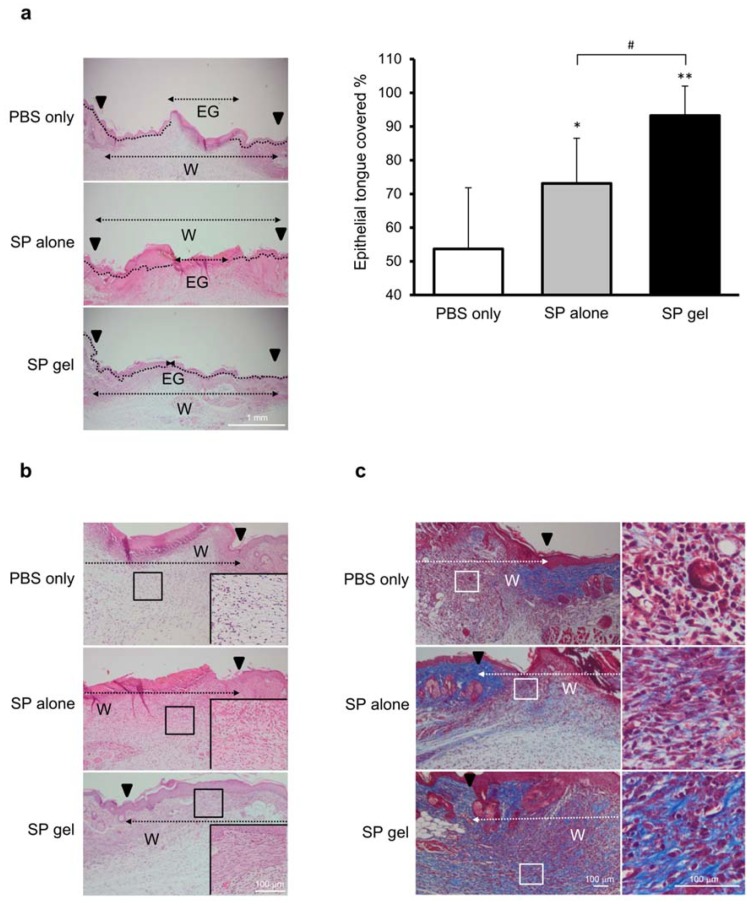
Histological analysis of SP gel-treated wounds. (**a**) Quantification of epithelial tongue coverage. The wound edge (W) is indicated by a black arrowhead in histological sections. The epithelial gap (EG), which is the distance between the edges of the epithelium, is indicated by a black dotted line. Values represent mean ± SD (*n* = 13 for PBS alone, *n* = 8 for SP alone, and *n* = 5 for SP gel). * *p* < 0.05, ** *p* < 0.01 vs. PBS-only negative control; ^#^ *p* < 0.05 vs. SP alone. (**b**,**c**) Granulation tissue formation and collagen synthesis induced by treatment with SP gel. Histological sections of wounds were stained with H&E (**b**) or Masson’s trichrome (**c**) on day 6. Higher magnifications are shown in the inset in the lower right corner (**b**) and in panels to the right (**c**).

To evaluate the clinical applicability of the SP gel, we assessed the in vivo wound healing activity of SP gel in a mouse excisional wound splinting model (Figure 5 and Figure 6). Ring-shaped silicone splints were applied to the skin 1 mm away from the wounds, and the wound closure activity was evaluated by measuring the wound area (%). At 6 days post-injury, the area of the wound treated with SP gel was significantly smaller (17.5% of the original area) than that of wounds treated with SP alone or PBS (43.6% and 55.8%, respectively, of the original area) (*p* < 0.01). Interestingly, the difference in area between wounds treated with SP gel and SP alone was statistically significant (Figure 5c,d) (*p* < 0.05). There was no difference in the area of wounds treated with PBS or gel without SP (data not shown).

To evaluate the progression of wound healing on day 6, we measured the area of the epithelial tongue covering the wound. The area of re-epithelialization was larger for wounds treated with SP gel as compared to that for wounds treated with either SP alone (*p* < 0.05) or PBS (*p* < 0.01) (Figure 6a), with a statistically significant difference between the SP gel and SP only groups (*p* < 0.05).

H&E and Masson’s trichrome staining revealed a higher density of granulation tissue, fibroblasts, and collagen in the SP gel treatment group as compared to the SP or PBS only groups (Figure 6b,c). Specifically, SP gel-treated groups showed well-formed granulation tissue with compact, oriented collagen deposition and covered by newly formed epidermal layer. Fibroblast proliferation and collagen deposition were observed in the SP gel-treated groups, whereas the granulation tissue was not fully formed in PBS or SP only groups, with weak collagen deposition. 

## 3. Discussion

Peptides are readily broken down by proteases under physiological conditions [23,24], and are oxidized by atmospheric oxygen, especially at higher temperature [25]. Various approaches including chemical alteration [26,27] and modification of natural peptides such as non-natural amino acid substitution [28,29] have been employed to protect biologically active peptides from degradation; however, structural modifications can often impede their biological activity [30]. Moreover, it is unclear to what extent a functional peptide with natural amino acids can be stabilized solely by sequence optimization [26]. Therefore, other methods for improving peptide stability must be considered.

In this study, we developed a novel SP gel formulation that is more stable than SP alone. We tested the stability of SP in SP gel in growth medium containing fetal bovine serum and at a range of temperatures by ELISA using an anti-SP antibody that binds to intact SP without cross-reacting with degraded and oxidized forms of the peptide (data not shown). We found that SP in SP gel was stable in FGM for up to 24 h (Figure S1) and at high temperatures (37 °C and 60 °C) for up to 4 weeks (Figure 2). The components of SP gel were selected based on previous studies reporting their positive effects on protein stability; these include sodium thiosulfate and polysorbate 80, which are anticipated to enhance the stability of SP by acting as free radical or oxygen scavengers that prevent protein oxidation [25] and minimizing physical damage to proteins from interfacial interaction caused by coating interfaces and/or protein-surfactant association [31,32,33]. We also included HEC, a gelling agent that is used in protein formulations to enhance viscosity and protein stability [34]. The optimized ratio of sodium thiosulfate and polysorbate 80 for SP gel was determined by analyzing SP stability (Figure 1). Mixtures containing ≥0.1% sodium thiosulfate and ≥0.006% polysorbate 80 had SP contents approaching 100%. Although these components were sufficient to stabilize SP, we added 1.5% HEC to the formulation to increase viscosity. HEC transformed the mixture into a hydrogel, which is a convenient and easy-to-use format for wound treatment.

SP gel showed more potential as a candidate wound-healing agent than SP alone, by stimulating, the proliferation and migration of HEKs and HDFs (Figure 3 and Figure 4). This is probably due to the fact that SP gel was more stable at the cell growth temperature of 37 °C than SP alone (Figure 2). SP gel also stimulated the closure of full-thickness wounds in mice to a greater extent than SP alone (Figure 5), with near-complete wound closure by day 6 (Figure 6). The mouse excisional wound splinting model allows an accurate assessment of wound healing because a splinting ring that tightly adheres to the skin surrounding the wound prevents wound closure by skin contraction, allowing wounds to heal instead through re-epithelialization [35,36]. In contrast, wounds treated with SP only or PBS showed a slightly effect on e-epithelialization. Our results suggest that the positive effects of SP gel on wound healing are due to the stability of SP in this formulation. Over 100 endogenous proteases are present in wound fluid [37] and contribute to various aspects of wound healing. However, SP in SP gel was highly stable in cell growth medium containing various proteases (Figure S1) and under different storage conditions (Figure 2). Thus, SP gel is useful from a therapeutic standpoint since it enhances wound healing while resisting degradation in vitro and in vivo.

Our results provide evidence for the benefits of SP gel as a topical agent for wound treatment. The work conducted in this study is important because (i) it is the first attempt to develop formulation of SP with improved stability, (ii) the novel formulation suggests some information applicable to formulation of other peptides with SP-like properties and finally (iii) SP gel could be a good candidate for clinical application to wound healing, owing to its beneficial characteristics, such as improved efficacy and stability. However, additional studies are required to clarify the mechanism of action of SP gel. It is possible that the wound healing stimulated by SP gel is mediated by regulation of associated cytokines. An earlier study showed that application of SP alone on an open wound promoted wound healing in rats via regulation of cytokines such as TGF-β1, TNF-α, and IL-10, among others [8]; accordingly, we are now confirming the regulation of cytokines by SP gel. We are also investigating the potential toxicity of SP gel; our preliminary results indicate that SP gel does not cause skin irritation in New Zealand white rabbits and is non-genotoxic in mice (data not shown). We expect that the market demand for SP gel will be high due to its efficacy, low cost, and improved stability over a range of temperatures, which make it a useful component of basic first aid kits, particularly in places where access to basic medical care is limited such as war zones and developing countries.

## 4. Conclusions

In addition to its stability, our novel SP formulation exhibited potent wound healing activities both in vitro and in vivo. SP gel was not degraded under various storage conditions and more effectively promoted wound healing than SP alone by inducing keratinocyte and fibroblast proliferation. Taken together, these results clearly demonstrate the benefits of SP gel as a promising topical agent for wound treatment.

## 5. Materials and Methods

### 5.1. Materials

All chemicals and reagents were purchased from Sigma-Aldrich (St. Louis, MO, USA) and were used as received unless otherwise indicated. Synthetic SP (RPKPQQFFGLM-NH_2_) was synthesized and purified to >85% by Anygen (Gwangju, Korea). The SP enzyme-linked immunosorbent assay (ELISA) kit was purchased from R&D Systems (Minneapolis, MN, USA). Human epidermal keratinocytes (HEKs) were cultured in keratinocyte growth medium (KGM; Lonza, Walkersville, MD, USA) and human dermal fibroblasts (HDFs) were cultured in fibroblast growth medium (FGM; Lonza).

### 5.2. Preparation of SP Gel

**Figure 7:**
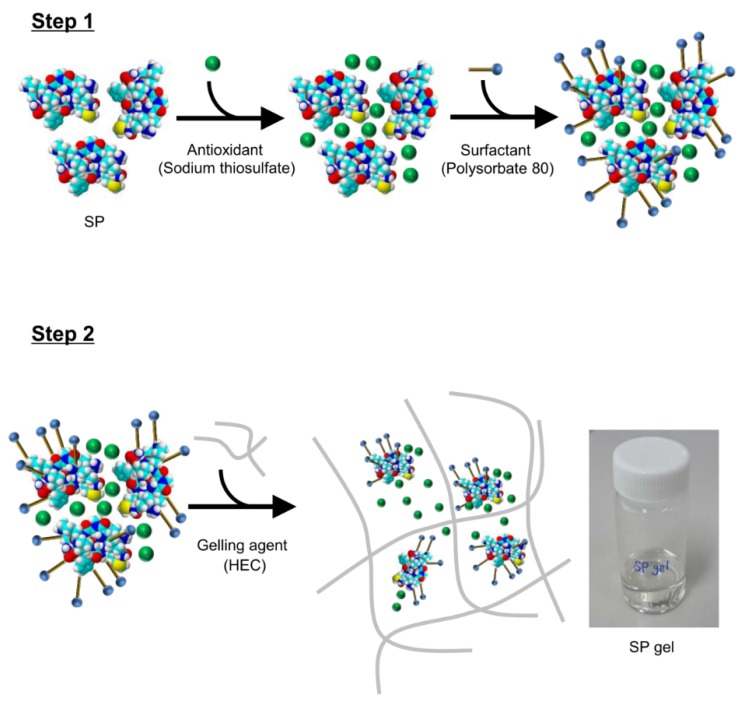
Schematic representation of SP gel formation for improved stability. SP, sodium thiosulfate, and polysorbate 80 are mixed together (Step 1) and then, HEC is added to the mixture, resulting in the formation of hydrogel networks (Step 2). SP is represented by space-filling models; green balls and blue sticks represent sodium thiosulfate and polysorbate 80, respectively, and gray lines represent HEC.

Lyophilized SP powder was first dissolved in phosphate-buffered saline (PBS). Sodium thiosulfate and polysorbate 80 were added followed by vortexing for 30 s to obtain a homogeneous solution. HEC was slowly added while rapidly stirring the solution until a translucent gel was formed [38] (Figure 7). The optimum ratio of sodium thiosulfate/polysorbate 80 in the SP formulation was determined by analyzing the concentration of SP (5 μg/mL) dissolved in PBS with sodium thiosulfate and polysorbate 80 using an SP ELISA kit. Experiments were performed at room temperature by incubating SP for 1 h with different concentrations of sodium thiosulfate (0.05–1%) and polysorbate 80 (0.003–0.1%). A sample without incubation at room temperature served as a control. HEC concentration of 1.5% in the SP gel was selected in accordance with a previous study [22].

### 5.3. Analysis of SP Stability in Gel

The stability of SP in the SP gel was evaluated at different temperatures. SP (5 μg/mL) in PBS (SP alone) or in the gel form (SP gel) were stored at different temperatures (60 °C, 37 °C, room temperature, or 4 °C) for 4 weeks. Aliquots of sample were removed at the indicated times and diluted with PBS for analysis of SP content by ELISA. A sample without 4-week incubation period served as a control.

### 5.4. Isolation and Culture of HEKs and HDFs

Primary HEKs and HDFs were isolated from human foreskin biopsy samples provided by Chung-Ang University Hospital in Korea [IRB No. C2014234(1431)]. Immediately before the experiments, samples were washed three times with PBS solution and the subcutaneous fatty tissue and blood vessels were excised with a sharp blade. The resultant tissue sheets were cut into small pieces that were transferred to 0.5% dispase II solution and incubated at 37 °C for 2 h. The epidermis and dermis were separated using forceps and incubated with 0.05% trypsin in PBS for 30 min and 0.2% collagenase solution for 2 h, respectively. After inhibiting trypsin and collagenase activities by adding 10% fetal bovine serum, each tissue fragment was filtered through a cell strainer and washed in PBS. The filtered HEKs or HDFs were seeded on plastic dishes and cultured in KGM and FGM, respectively.

### 5.5. Cell Proliferation Assay

HEKs and HDFs were separately seeded in 96-well Nunc plates (Nalgene, Rochester, NY, USA) at a density of 3 × 10^4^ cells/well in 0.1 mL of culture medium and allowed to attach overnight. Culture medium containing SP alone or SP gel (final SP concentrations ranging from 1 to 10 μg/mL) was added to the wells, with medium containing PBS only used as a negative control. After incubation for 24 h, 10 μL of 3-(4,5-dimethylthiazol-2-yl)-2,5-diphenyl tetrazolium bromide (MTT, 5 mg/mL in PBS; Promega, Madison, WI, USA) were added to each well followed by incubation for 3 h at 37 °C. After removing the medium, the formazan crystals were dissolved in isopropanol and the optical density at 570 nm was measured on a VERSAmax Tunable microplate reader (Molecular Devices, Sunnyvale, CA, USA). The percentage of cell proliferation was determined using the following equation: Relative cell proliferation (%) = [(A_s_ − A_0_)/(A_c_ − A_0_)] × 100, where A_s_ is the absorbance of the sample, A_c_ is the absorbance of negative control, and A_0_ is the background absorbance. At least three independent experiments were performed.

### 5.6. Cell Migration Assay

In vitro cell migration in confluent cell monolayers was evaluated as previously described [39]. HEKs and HDFs were separately seeded in 6-well Nunc plates and grown until they reached 100% confluence (48 h). An artificial wound was created by scratching the bottom of the plate with a 200-μL pipette tip. After washing twice with PBS to remove cellular debris, cells were treated with culture medium containing SP alone or SP gel, with medium containing PBS only serving as a negative control. Cells were photographed before (0 h) and 24 h after peptide treatment on an inverted phase-contrast microscope (Olympus, Tokyo, Japan). Images were captured and wound areas were estimated using Photoshop v.5.0 software (Adobe Systems, San Jose, CA, USA). Cell migration rate was calculated as [(X_0h_ − X_24h_)/(C_0h_ − C_24h_)] × 100, where X_0h_ and X_24h_ are the areas of the scratch at 0 and 24 h exposure of the samples, respectively; and C_0h_ and C_24h_ are the areas of the scratch at 0 and 24 h exposure to the PBS, respectively, after exposure to the negative control. At least three independent experiments were performed.

### 5.7. In Vivo Wound Healing Assay

The animal experimental protocol followed the Guide for the Care and Use of Laboratory Animals published by the US National institutes of Health (NIH Publication No. 80-23). All animal study experiments were performed in accordance with the Animal Care and Use Committee of Kyung Hee University. Female BALB/c mice (7 weeks old) were purchased from Doo-Yeol Biotech (Seoul, Korea). After acclimatization for 7 days, the mice were anesthetized and the hair on their dorsal surface was shaved. Two full-thickness wounds were made to the back of each mouse using a 5-mm biopsy punch as previously described [35]. After creating the wounds, ring-shaped silicone splints (inner diameter = 8 mm, outer diameter = 12 mm) fabricated from 0.5 mm-thick silicone sheets (Grace Bio-Labs, Bend, OR, USA) were applied to the skin 1 mm away from the wound perimeter and were affixed with an instant-bonding cyanoacrylate adhesive (Krazy Glue, Columbus, OH, USA) and four interrupted stitches made using 4-0 silk sutures. A 30-μL volume of SP alone (*n* = 8) or SP gel (*n* = 5) was applied to the wound area every 24 h for 5 days. Equal amount of SP (5 μg/mL) was used when applying either SP alone or aSP gel. The wounds on the opposite side were treated with 30 μL PBS (negative control) (*n* = 13). The wounds were covered with a transparent film dressing (Tegaderm; 3M Health Care, St. Paul, MN, USA). The mice were sacrificed 6 days after injury; wound sites were digitally photographed, and wound areas were determined from the images using Photoshop v.5.0 software. Changes in wound area over time are expressed as real wound area divided by splinted hole area.

Explants were fixed and dehydrated in a graded series of ethanol (70–100%), embedded in paraffin, and serially sectioned at a thickness of 5 μm. The sections were treated with hematoxylin and eosin (H&E) or Masson’s trichrome stain. Images were acquired using a BX41 light microscope (Olympus) and were analyzed using Photoshop v.5.0 software. The percentage of epithelial tongue coverage was calculated as [Wound edge (W) − Epithelial gap (EG)/wound edge (W)] × 100. The epithelial tongue was quantified as previously described [40].

### 5.8. Statistical Analysis

Data are reported as mean ± SD and were analyzed with the Student’s *t* test. *p* < 0.05 was considered statistically significant.

## Figures and Tables

**Table 1:** SP gel formulation.

Component	Amount	Expected Function
Sodium thiosulfate	0.1%	SP stability
Polysorbate 80	0.006%	
HEC	1.5%	Viscosity
SP	Indicated amount	Wound healing

## Supplementary Materials

The supplementary materials are available online.
