# Supplementary material for: A Novel Substance P-Based Hydrogel for Increased Wound Healing Efficiency
Source: Molecules. 2018 Aug 31;23(9):2215. doi: 10.3390/molecules23092215 (PMC6225281; doi:10.3390/molecules23092215)
Supplement: Supplementary file 1 [file molecules-23-02215-s001.pdf]

Supplemental information

Supplementary materials and methods

Analysis of SP stability in gel in cell growth medium FGM

The stability of SP in the SP gel was analyzed in the fibroblast growth medium, FGM. SP (5 μg/ml) in PBS (SP alone) or in the gel form (SP gel) were stored in FGM at 37°C for up to 24 hours. Aliquots of the sample were removed at the indicated times and diluted in PBS for analysis of SP content by ELISA. A sample without incubation in FGM served as control.

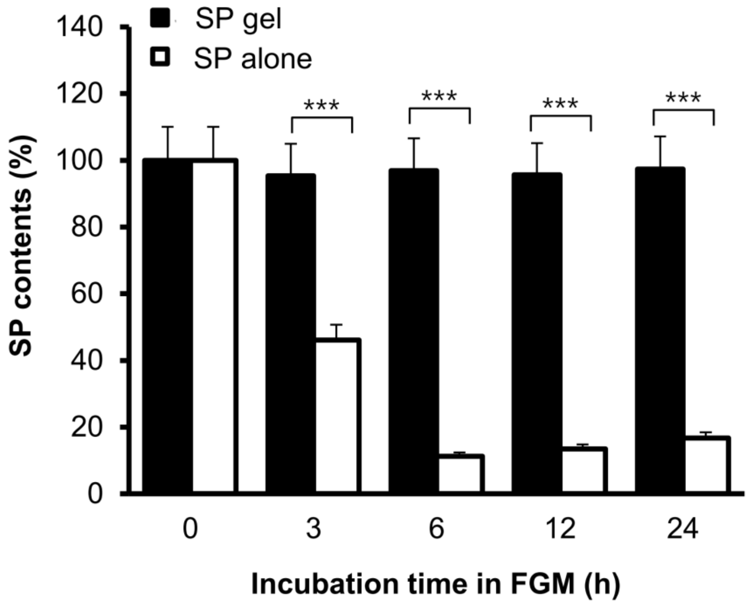

Figure S1. SP gel stability in FGM. SP alone or SP gel was stored in cell growth medium FGM for up to 24 hours. A sample at 0 hours without incubation in FGM served as control. The stability of SP alone and SP gel was analyzed by ELISA. Values represent mean ± SD of three independent experiments. ***P < 0.001.
